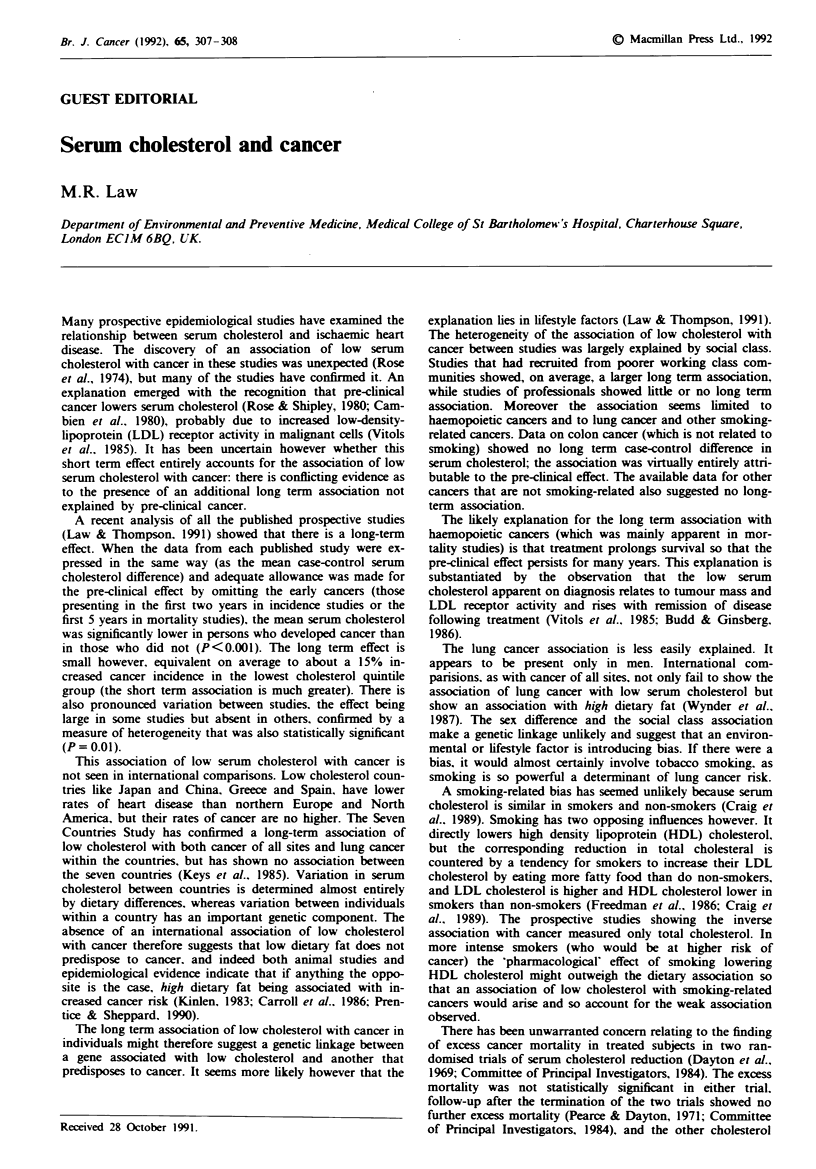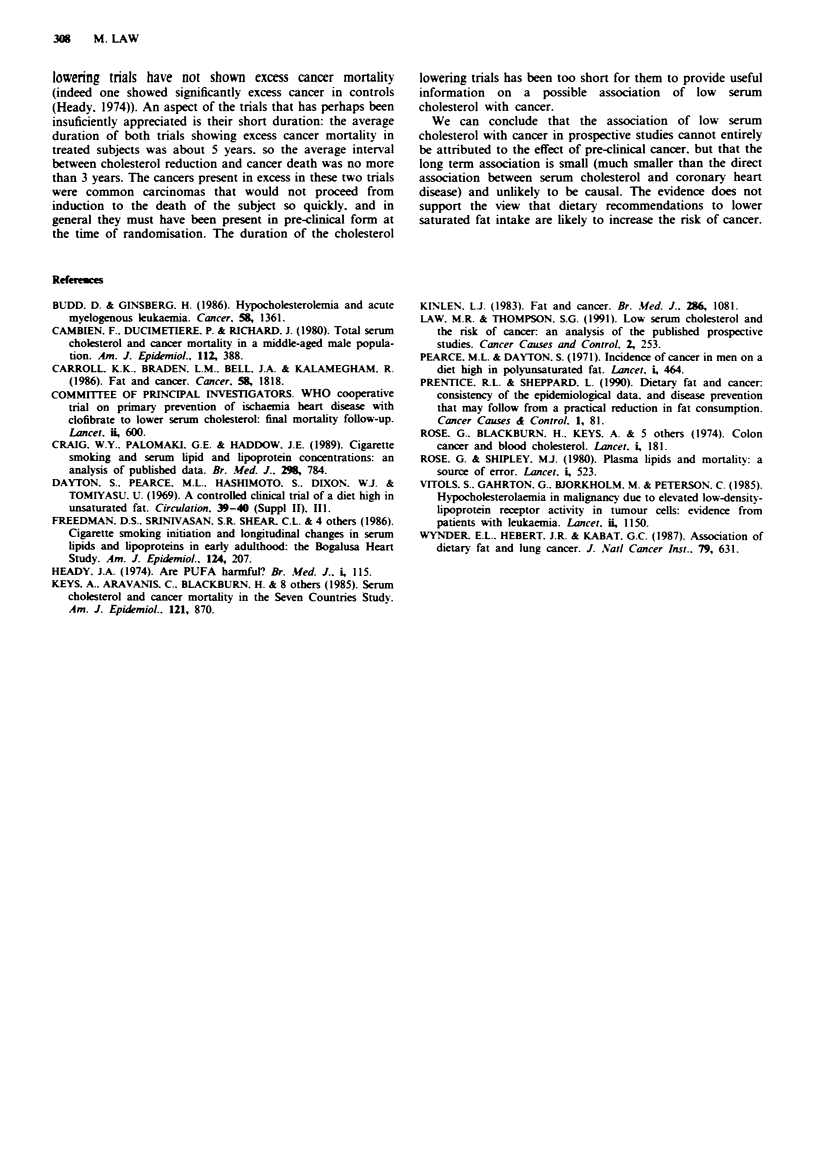# Serum cholesterol and cancer.

**DOI:** 10.1038/bjc.1992.63

**Published:** 1992-03

**Authors:** M. R. Law


Br. J. Cancer (1992), 65, 307-308 © Macmillan Press Ltd., 1992

GUEST EDITORIAL

### Serum cholesterol and cancer

M.R. Law

*Department of Environmental and Preventive Medicine, Medical College of St Bartholomew's Hospital, Charterhouse Square, London EC1M 6BQ, UK.*

Many prospective epidemiological studies have examined the relationship between serum cholesterol and ischaemic heart disease. The discovery of an association of low serum cholesterol with cancer in these studies was unexpected (Rose *et al.*, 1974), but many of the studies have confirmed it. An explanation emerged with the recognition that pre-clinical cancer lowers serum cholesterol (Rose & Shipley, 1980; Cambien *et al.*, 1980), probably due to increased low-density-lipoprotein (LDL) receptor activity in malignant cells (Vitols *et al.*, 1985). It has been uncertain however whether this short term effect entirely accounts for the association of low serum cholesterol with cancer: there is conflicting evidence as to the presence of an additional long term association not explained by pre-clinical cancer.

A recent analysis of all the published prospective studies (Law & Thompson, 1991) showed that there is a long-term effect. When the data from each published study were expressed in the same way (as the mean case-control serum cholesterol difference) and adequate allowance was made for the pre-clinical effect by omitting the early cancers (those presenting in the first two years in incidence studies or the first 5 years in mortality studies), the mean serum cholesterol was significantly lower in persons who developed cancer than in those who did not ($P<0.001$). The long term effect is small however, equivalent on average to about a 15% increased cancer incidence in the lowest cholesterol quintile group (the short term association is much greater). There is also pronounced variation between studies, the effect being large in some studies but absent in others, confirmed by a measure of heterogeneity that was also statistically significant ($P=0.01$).

This association of low serum cholesterol with cancer is not seen in international comparisons. Low cholesterol countries like Japan and China, Greece and Spain, have lower rates of heart disease than northern Europe and North America, but their rates of cancer are no higher. The Seven Countries Study has confirmed a long-term association of low cholesterol with both cancer of all sites and lung cancer within the countries, but has shown no association between the seven countries (Keys *et al.*, 1985). Variation in serum cholesterol between countries is determined almost entirely by dietary differences, whereas variation between individuals within a country has an important genetic component. The absence of an international association of low cholesterol with cancer therefore suggests that low dietary fat does not predispose to cancer, and indeed both animal studies and epidemiological evidence indicate that if anything the opposite is the case, *high* dietary fat being associated with increased cancer risk (Kinlen, 1983; Carroll *et al.*, 1986; Prentice & Sheppard, 1990).

The long term association of low cholesterol with cancer in individuals might therefore suggest a genetic linkage between a gene associated with low cholesterol and another that predisposes to cancer. It seems more likely however that the explanation lies in lifestyle factors (Law & Thompson, 1991). The heterogeneity of the association of low cholesterol with cancer between studies was largely explained by social class. Studies that had recruited from poorer working class communities showed, on average, a larger long term association, while studies of professionals showed little or no long term association. Moreover the association seems limited to haemopoietic cancers and to lung cancer and other smoking-related cancers. Data on colon cancer (which is not related to smoking) showed no long term case-control difference in serum cholesterol; the association was virtually entirely attributable to the pre-clinical effect. The available data for other cancers that are not smoking-related also suggested no long-term association.

The likely explanation for the long term association with haemopoietic cancers (which was mainly apparent in mortality studies) is that treatment prolongs survival so that the pre-clinical effect persists for many years. This explanation is substantiated by the observation that the low serum cholesterol apparent on diagnosis relates to tumour mass and LDL receptor activity and rises with remission of disease following treatment (Vitols *et al.*, 1985; Budd & Ginsberg, 1986).

The lung cancer association is less easily explained. It appears to be present only in men. International comparisions, as with cancer of all sites, not only fail to show the association of lung cancer with low serum cholesterol but show an association with *high* dietary fat (Wynder *et al.*, 1987). The sex difference and the social class association make a genetic linkage unlikely and suggest that an environmental or lifestyle factor is introducing bias. If there were a bias, it would almost certainly involve tobacco smoking, as smoking is so powerful a determinant of lung cancer risk.

A smoking-related bias has seemed unlikely because serum cholesterol is similar in smokers and non-smokers (Craig *et al.*, 1989). Smoking has two opposing influences however. It directly lowers high density lipoprotein (HDL) cholesterol, but the corresponding reduction in total cholesteral is countered by a tendency for smokers to increase their LDL cholesterol by eating more fatty food than do non-smokers, and LDL cholesterol is higher and HDL cholesterol lower in smokers than non-smokers (Freedman *et al.*, 1986; Craig *et al.*, 1989). The prospective studies showing the inverse association with cancer measured only total cholesterol. In more intense smokers (who would be at higher risk of cancer) the 'pharmacological' effect of smoking lowering HDL cholesterol might outweigh the dietary association so that an association of low cholesterol with smoking-related cancers would arise and so account for the weak association observed.

There has been unwarranted concern relating to the finding of excess cancer mortality in treated subjects in two randomised trials of serum cholesterol reduction (Dayton *et al.*, 1969; Committee of Principal Investigators, 1984). The excess mortality was not statistically significant in either trial, follow-up after the termination of the two trials showed no further excess mortality (Pearce & Dayton, 1971; Committee of Principal Investigators, 1984), and the other cholesterol

Received 28 October 1991.

lowering trials have not shown excess cancer mortality (indeed one showed significantly excess cancer in controls (Heady, 1974)). An aspect of the trials that has perhaps been insuficiently appreciated is their short duration: the average duration of both trials showing excess cancer mortality in treated subjects was about 5 years, so the average interval between cholesterol reduction and cancer death was no more than 3 years. The cancers present in excess in these two trials were common carcinomas that would not proceed from induction to the death of the subject so quickly, and in general they must have been present in pre-clinical form at the time of randomisation. The duration of the cholesterol lowering trials has been too short for them to provide useful information on a possible association of low serum cholesterol with cancer.

We can conclude that the association of low serum cholesterol with cancer in prospective studies cannot entirely be attributed to the effect of pre-clinical cancer, but that the long term association is small (much smaller than the direct association between serum cholesterol and coronary heart disease) and unlikely to be causal. The evidence does not support the view that dietary recommendations to lower saturated fat intake are likely to increase the risk of cancer.

### References

BUDD, D. & GINSBERG, H. (1986). Hypocholesterolemia and acute myelogenous leukaemia. *Cancer*, **58**, 1361.

CAMBIEN, F., DUCIMETIERE, P. & RICHARD, J. (1980). Total serum cholesterol and cancer mortality in a middle-aged male population. *Am. J. Epidemiol.*, **112**, 388.

CARROLL, K.K., BRADEN, L.M., BELL, J.A. & KALAMEGHAM, R. (1986). Fat and cancer. *Cancer*, **58**, 1818.

COMMITTEE OF PRINCIPAL INVESTIGATORS. WHO cooperative trial on primary prevention of ischaemia heart disease with clofibrate to lower serum cholesterol: final mortality follow-up. *Lancet*, **ii**, 600.

CRAIG, W.Y., PALOMAKI, G.E. & HADDOW, J.E. (1989). Cigarette smoking and serum lipid and lipoprotein concentrations: an analysis of published data. *Br. Med. J.*, **298**, 784.

DAYTON, S., PEARCE, M.L., HASHIMOTO, S., DIXON, W.J. & TOMIYASU, U. (1969). A controlled clinical trial of a diet high in unsaturated fat. *Circulation*, **39–40** (Suppl II), II1.

FREEDMAN, D.S., SRINIVASAN, S.R. SHEAR, C.L. & 4 others (1986). Cigarette smoking initiation and longitudinal changes in serum lipids and lipoproteins in early adulthood: the Bogalusa Heart Study. *Am. J. Epidemiol.*, **124**, 207.

HEADY, J.A. (1974). Are PUFA harmful? *Br. Med. J.*, **i**, 115.

KEYS, A., ARAVANIS, C., BLACKBURN, H. & 8 others (1985). Serum cholesterol and cancer mortality in the Seven Countries Study. *Am. J. Epidemiol.*, **121**, 870.

KINLEN, L.J. (1983). Fat and cancer. *Br. Med. J.*, **286**, 1081.

LAW, M.R. & THOMPSON, S.G. (1991). Low serum cholesterol and the risk of cancer: an analysis of the published prospective studies. *Cancer Causes and Control*, **2**, 253.

PEARCE, M.L. & DAYTON, S. (1971). Incidence of cancer in men on a diet high in polyunsaturated fat. *Lancet*, **i**, 464.

PRENTICE, R.L. & SHEPPARD, L. (1990). Dietary fat and cancer: consistency of the epidemiological data, and disease prevention that may follow from a practical reduction in fat consumption. *Cancer Causes & Control*, **1**, 81.

ROSE, G., BLACKBURN, H., KEYS, A. & 5 others (1974). Colon cancer and blood cholesterol. *Lancet*, **i**, 181.

ROSE, G. & SHIPLEY, M.J. (1980). Plasma lipids and mortality: a source of error. *Lancet*, **i**, 523.

VITOLS, S., GAHRTON, G., BJORKHOLM, M. & PETERSON, C. (1985). Hypocholesterolaemia in malignancy due to elevated low-density-lipoprotein receptor activity in tumour cells: evidence from patients with leukaemia. *Lancet*, **ii**, 1150.

WYNDER, E.L., HEBERT, J.R. & KABAT, G.C. (1987). Association of dietary fat and lung cancer. *J. Natl Cancer Inst.*, **79**, 631.